# The Direct Healthcare Expenditures of Prostate Cancer by Disease Severity: Evidence from US National Survey Data

**DOI:** 10.36469/9844

**Published:** 2016-10-28

**Authors:** Peter J. Mallow, Jie Chen, John A. Rizzo, John R. Penrod, Geralyn C. Trudel, Teresa M. Zyczynski

**Affiliations:** 1 CTI Clinical Trial & Consulting Services, Cincinnati, OH, USA; 2 University of Maryland, College Park, MD, USA; 3 Stony Brook University, Stony Brook, NY, USA; 4 Bristol-Myers Squibb, Princeton, NJ, USA; 5 Bristol-Myers Squibb, Montréal, QC, Canada

**Keywords:** prostate cancer, health economics, disease severity, direct health care costs

## Abstract

**Background:** In the United States, approximately 2.8 million men have a history of prostate cancer (PC).

**Objective:** This study quantified the effects of PC, overall and by disease severity on direct healthcare costs to insurers and patients.

**Methods:** Using 1996–2010 data from the Medical Expenditure Panel Survey (MEPS), a large, nationally representative US database, multivariate analyses were used to assess the relationship between PC and direct annual healthcare costs to insurers and patients, at individual and US aggregate levels. Men aged 40 years and older with International Classification of Diseases, Ninth Revision (ICD-9) diagnosis code 185 were identified. Disease severity was determined with clinical assistance and based, in part, on the data in MEPS. The cohorts were: localized cancer not treated with chemotherapy, localized cancer treated with chemotherapy, and metastatic cancer.

**Results:** The MEPS database included 1297 patients with PC: 811 patients with localized PC not treated with chemotherapy, 426 patients with PC treated with chemotherapy, and 60 patients with metastatic PC. PC had a larger effect on incremental costs for metastatic patients, $20 357, vs $16 709 for localized PC with chemotherapy, and $5238 for localized PC with no chemotherapy. When aggregated to the US population, PC accounted for an incremental annual cost of $15 billion. The largest aggregate annual costs were incurred by patients with localized PC treated with chemotherapy ($8.6 billion), compared to those not treated with chemotherapy ($4.8 billion) and metastatic patients ($1.6 billion).

**Conclusions:** The aggregate annual costs of PC are substantial for all groups examined and greatest for patients with localized cancer treated with chemotherapy. This reflects the relatively high prevalence and high per capita healthcare expenditures associated with this group. With a growing and aging population, the prevalence of PC is expected to rise, increasing the burden on public health.

## BACKGROUND

Approximately 2.8 million men in the United States have a history of prostate cancer (PC), making it the most common cancer in men.[1] It is surpassed only by lung cancer as a leading cause of death from cancer in men.[2,3] An estimated 233 000 new cases of prostate cancer are expected in 2014 in the United States, and 29 480 men will die from the disease.[3]

Earlier detection via serum prostate-specific antigen (PSA) screening has helped in monitoring disease progression and identifying men who might benefit from treatment. Thus, many men are now diagnosed sooner, usually with lower-stage cancer than previously. This has improved the accuracy of PC prevalence estimates, allowing for better assessments of the economic burden of this disease.[4] However, despite advances in early detection and treatment, the disease remains one of the leading causes of cancer-related mortality among American men.[2,3]

The high prevalence and mortality from PC suggests that the costs of this disease are large, though available evidence on direct healthcare expenditures related to PC is limited. Prior studies have focused on samples of PC patients that are either small, not representative (by geography or age), or have employed rough proxies for disease severity.[5-8] One study stratified subjects by time from initial diagnosis or whether they were in their last year of life as a crude proxy for severity.[7] The study found that the average annual net cost of care for PC ranges from $3201 for continuing care to $93 363 in the last year of life (2010 US dollars).[7]

To improve understanding of the direct economic cost of PC in the United States, this study examined healthcare expenditures for PC using a sample of patients that is nationally representative. We also estimated how these costs vary by disease severity.

## METHODS

A retrospective analysis was conducted using the Medical Expenditure Panel Survey (MEPS), a large, nationally representative database developed by the Agency for Healthcare Research and Quality (AHRQ), to quantify individual and US national estimates of the healthcare insurer expenditures and patient out-of-pocket (OOP) expenditures associated with PC for three levels of disease severity: localized cancer not treated with chemotherapy, localized cancer treated with chemotherapy, and metastatic cancer.

### Data

This study used data from the 1996–2010 MEPS, a subset of the National Health Interview Survey (NHIS), which includes information on healthcare utilization and expenditures, health status, health insurance coverage, and socio-demographic and socio-economic characteristics for the civilian, non-institutionalized population in the United States.[9] MEPS utilizes a complex survey design which includes clustering and oversampling of certain subgroups, such as minorities.[10] MEPS collects comprehensive data on individuals and their healthcare expenditures and use over a span of roughly 2 years, with response rates varying from approximately 60–80%. Household survey data are collected by means of computer-assisted personal interviews, with data supplemented by information collected directly from the medical providers used by survey participants. Insurance data are collected both from households and through a separate survey of employers' business establishments, which collects information on health insurance provided by US employers. Healthcare expenditure data in MEPS are self-reported; however, medical providers help validate the self-reported data and resolve inconsistencies when they occur.

The sample included in this study consisted of men who were at least 40 years of age and had an International Classification of Diseases, Ninth Revision (ICD-9) diagnosis code of 185 (malignant neoplasm of prostate). Because we sought to examine the relative importance of OOP expenditures compared to insurer expenditures, the sample excluded uninsured subjects. Expenditure estimates were generated for subjects based on three levels of disease severity: localized cancer not treated with chemotherapy, localized cancer treated with chemotherapy, and metastatic cancer. These severity-based results were then combined to generate aggregate estimates.

The division of PC disease severity into categories for this analysis was determined, in part, by the types of treatment data that were captured in the MEPS database. The available data allowed identification of patients with metastases and patients who had received any of a list of drugs that were identified as specific for chemotherapeutic treatment of PC. These data elements, along with diagnoses of other cancers and their start dates that were available in the database, were used to assign patients to categories of PC severity. While the resulting three categories of disease severity do not fully measure disease stage, they do capture three distinct states of disease progression, from mild to advanced, thus providing a reasonable proxy measure for disease severity. The full schema used to define severity of PC is presented in Figure 1.

**Figure 1.** PC Severity Definitions

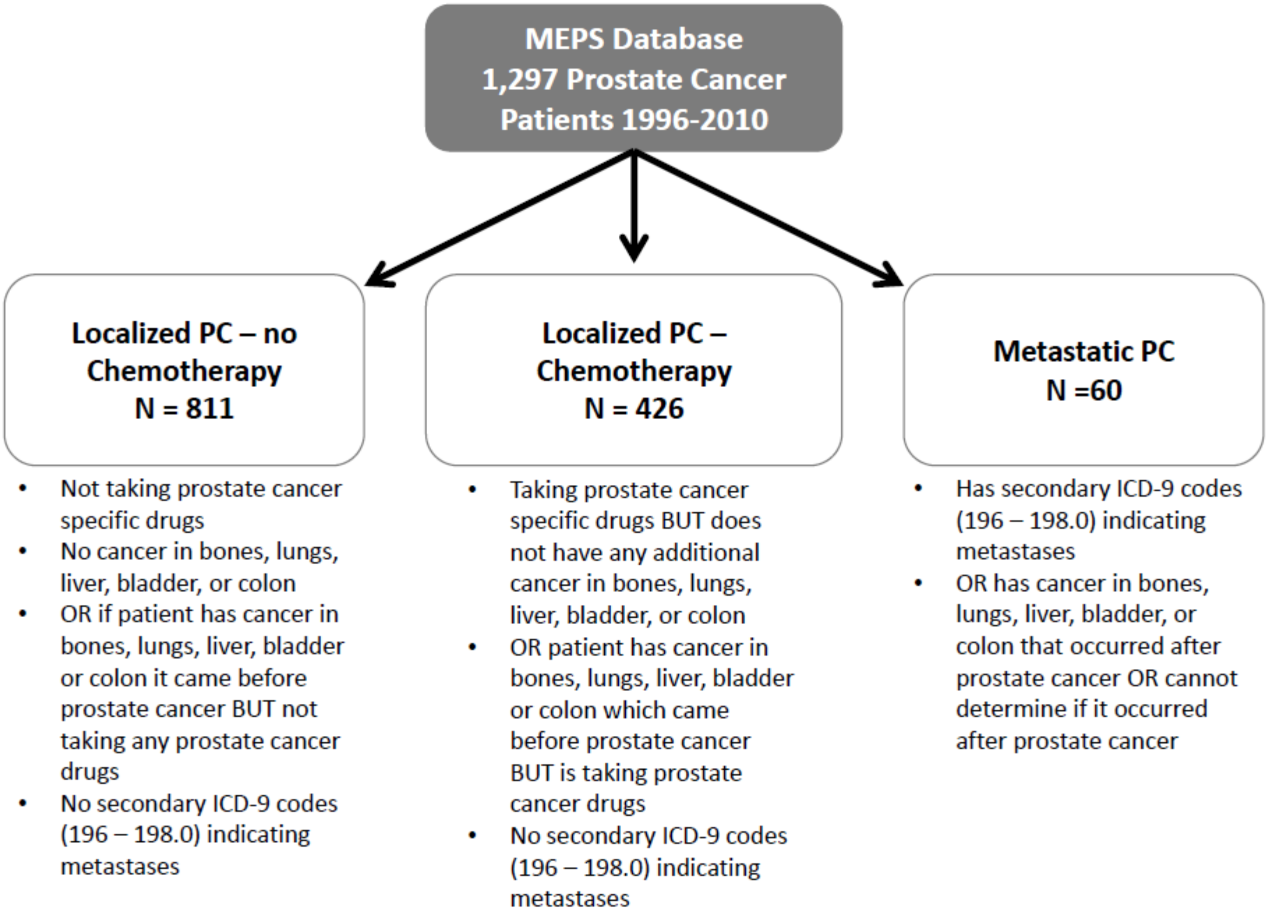

MEPS: Medical Expenditure Panel Survey; PC: prostate cancer; ICD-9: International Classification of Diseases, 9th Revision

### Dependent Variable: Medical Expenditures

The expenditure data contained in MEPS included spending on physicians, hospital and outpatient services, medications, diagnostic testing, and other medical services. This study used total annual expenditures on these various healthcare services, and categorized these total expenditures according to the amounts paid by health insurers and patients' OOP payments.

### Explanatory Variables: Clinical and Socio-demographic Characteristics

This study included a number of predictors of expenditures, including major chronic diseases, socio-demographics, geographic region, and year. Chronic diseases were measured as binary variables (1 = disease is present; 0 = disease is absent) and included PC along with 30 other major chronic illnesses. These diseases were chosen based on their prevalence and clinical considerations. A wide array of comorbidities were included because previous research has shown that failure to control adequately for comorbidities may lead to substantial upward bias in the estimated expenditure impact of the disease of interest.[11]

Socio-demographic factors included age strata, education, race, income, marital status, and health insurance type. Race variables included African American, Hispanic, and other race/ethnicity, with White serving as the reference group. Insurance status was measured as a series of binary indicators: Medicaid, Medicare, other public insurance, private non-health maintenance organization (HMO) insurance, and private HMO insurance (reference group). Geographic variables included Census Region (Midwest, South, West, and Northeast, with Northeast serving as the reference region) and whether a patient resided in a Metropolitan Statistical Area (MSA). Year was measured as a series of binary variables, with 1996 serving as the reference year.

### Statistical Methods

Two-part models were estimated in which the likelihood of incurring any expenditures and the conditional expenditures were estimated separately by logistic and ordinary least squares (OLS) models, respectively.[12] Specifically, the analysis estimated the likelihood that a patient had positive medical expenditures, and separate conditional expenditure models were constructed for OOP expenses and insurer expenditures. The two-part model is frequently used in health economics research when many observations are clustered and the remaining observations are skewed to the right.[13] To normalize the distribution of the error terms, expenditures were log-transformed.

The logistic regression models were written as:

$$(1)\quad PrEXPENDITURES = a_0 + a_1\, PCANCER_{SEV1} + a_2\, PCANCER_{SEV2} + a_3\, PCANCER_{SEV3} + \beta\, Comorbidities + \Theta X + \varepsilon,$$

where *PrEXPENDITURES* is a binary variable equal to 1 if medical expenses are positive and 0 otherwise; $PCANCER_{SEVi}(i = 1,2,3)$ are binary variables capturing prostate disease severity, where subscript = 1 indicates least severe (i.e. localized PC not treated with chemotherapy) and 3 is most severe (i.e. metastatic PC); Comorbidities are binary indicator variables for the presence or absence of other diseases; $X$ is a vector of socio-demographic, economic, region, and year variables; $a_0$, $a_1$, $\beta$, and $\Theta$ are coefficients to be estimated; and ε is the error term. Equation (1) was estimated separately to ascertain the probabilities of healthcare insurer and OOP expenditures. The effects of each PC severity cohort on costs are measured relative to male subjects without PC, who serve as the reference group.

In the second stage, conditional expenditure models were estimated by the following equation:

$$(2)\quad lnEXPENDITURES = a_0 + a_1\, PCANCER_{SEV1} + a_2\, PCANCER_{SEV2} + a_3\, PCANCER_{SEV3} + \beta\, Comorbidities + \Theta X + \varepsilon,$$

where $lnEXPENDITURES$ is the natural logarithm of conditional healthcare expenditures and other terms are as defined above. The models were again estimated separately for healthcare insurer and OOP expenditures. Expenditures were adjusted to 2011 US dollars using the Medical Care component of the Consumer Price Index (CPI). All models were estimated using STATA version 11 (STATA®, STATA Corporation, LP, College Station, TX USA).

**Sensitivity Analysis**

The effects of disease on the burden of illness are sensitive to model specifications and disease prevalence. Additionally, patients with more severe disease may be systematically more likely to indicate the presence of disease. Thus, using the self-reported disease measure may bias incremental expenditures estimates upward. On the other hand, random measurement error in the disease indicator variable may bias the incremental expenditures estimates downward.[14] Therefore, to address these sources of uncertainty and to gauge the robustness of the results to alternative assumptions, sensitivity analyses were performed by varying the prevalence rate of PC from baseline ± 25%. Also, the estimated incremental expenditure effects of PC were varied by 10% above and below for each prevalence rate. For the baseline value, the prevalence rate for PC in MEPS was used, which is within the range reported in the literature.[7,15]

## RESULTS

The total sample included 1297 men with PC and 61,062 without PC. The cohort of patients with localized PC not treated with chemotherapy cohort comprised 62.54% (n = 811) of the PC sample. The cohort of patients with localized PC treated with chemotherapy cohort and the cohort with metastatic PC comprised 32.84% (n = 426) and 4.62% (n = 60), respectively of the PC sample. Mean annual healthcare insurer expenditures were $10 115, $15 048, and $23 072 in the localized no chemotherapy, localized with chemotherapy and metastatic PC cohorts, respectively. The mean annual healthcare insurer expenditure was $6,262 for the cohort without PC (Table 1). Mean annual OOP expenditures were also higher in all three PC cohorts relative to the cohort without PC.

Patients with PC were more likely to have circulatory system diseases, emphysema, and metabolic diseases (Table 1). The presence of these comorbid conditions generally increased with the severity of the PC. Patients in the localized cancer with chemotherapy and metastatic PC cohorts were also more likely to be covered by Medicare.

The racial and ethnic composition of the localized PC with no chemotherapy cohort and the localized PC with chemotherapy cohort were comparable to the cohort without PC. In the metastatic PC cohort, 83% of the sample was white, substantially higher than the 70% to 72% composition of the other cohorts.

**Table 1.** Descriptive Statistics of Study Variables by PC Status for Direct Expenditures

| | Without PC n = 61 062 | Localized PC– no chemotherapy n =811 | | Localized PC – chemotherapy n = 426 | | Metastatic PC n = 60 | |
|---|---|---|---|---|---|---|---|
| | **Mean** | **Mean** | **p-Value** | **Mean** | **p-Value** | **Mean** | **p-Value** |
| **Dependent variables** | | | | | | | |
| Total insurer expenditures ($) if any | 6262 | 10 115 | <0.001 | 15 048 | <0.001 | 23 072 | <0.001 |
| Total OOP expenditures ($) if any | 1084 | 1539 | <0.001 | 2112 | <0.001 | 2168 | <0.001 |
| Probability of insurer expenditures | 0.92 | 0.98 | <0.001 | 1.00 | <0.001 | 1.00 | 0.02 |
| Probability of OOP expenditures | 0.92 | 0.97 | <0.001 | 0.99 | <0.001 | 0.98 | NS |
| Explanatory variables* (truncated)** | | | | | | | |
| **Medical condition** | | | | | | | |
| **Diseases of the circulatory system** | | | | | | | |
| Hypertension | 0.36 | 0.55 | <0.001 | 0.58 | <0.001 | 0.70 | <0.001 |
| CAD | 0.04 | 0.10 | <0.001 | 0.12 | <0.001 | 0.17 | <0.001 |
| CHF | 0.01 | 0.02 | NS | 0.04 | <0.001 | 0.05 | 0.02 |
| AMI | 0.03 | 0.07 | <0.001 | 0.05 | 0.03 | 0.15 | <0.001 |
| Stroke | 0.00 | 0.00 | NS | 0.00 | NS | 0.03 | <0.001 |
| Cardiac dysrhythmias | 0.03 | 0.06 | <0.001 | 0.07 | <0.001 | 0.03 | NS |
| **Diseases of the respiratory system** | | | | | | | |
| Chronic bronchitis | 0.01 | 0.02 | 0.02 | 0.03 | <0.001 | 0.02 | NS |
| Emphysema | 0.02 | 0.03 | 0.01 | 0.04 | <0.001 | 0.10 | <0.001 |
| Asthma | 0.04 | 0.03 | NS | 0.05 | NS | 0.02 | NS |
| **Metabolic diseases** | | | | | | | |
| Diabetes | 0.15 | 0.18 | 0.005 | 0.23 | <0.001 | 0.23 | NS |
| Hyperlipidemia | 0.25 | 0.40 | <0.001 | 0.37 | <0.001 | 0.53 | NS |
| Hypothyroidism | 0.01 | 0.03 | <0.001 | 0.02 | NS | 0.02 | NS |
| **Age (years)** | | | <0.001 | | <0.001 | | <0.001 |
| 40–64 | 0.69 | 0.26 | | 0.24 | | 0.08 | |
| 65–74 | 0.18 | 0.36 | | 0.36 | | 0.48 | |
| ≥75 | 0.13 | 0.38 | | 0.40 | | 0.43 | |
| **Race/ethnicity** | | | <0.001 | | <0.001 | | <0.05 |
| White | 0.70 | 0.71 | | 0.72 | | 0.83 | |
| Hispanic | 0.13 | 0.09 | | 0.07 | | 0.05 | |
| African American | 0.12 | 0.17 | | 0.18 | | 0.12 | |
| Other | 0.05 | 0.03 | | 0.04 | | 0.00 | |
| **Health insurance** | | | <0.001 | | <0.001 | | <0.001 |
| Medicaid | 0.09 | 0.07 | | 0.11 | | 0.05 | |
| Medicare | 0.17 | 0.31 | | 0.34 | | 0.37 | |
| Other public health insurance | 0.01 | 0.01 | | 0.02 | | 0.03 | |
| Private non-HMO plan | 0.48 | 0.50 | | 0.54 | | 0.53 | |
| Private HMO plan | 0.31 | 0.17 | | 0.10 | | 0.08 | |

Table 1 footnotes

*Descriptive statistics for all explanatory variables are proportions.

**In the interest of brevity, other generally less common conditions are not reported in Table 1. Variables measuring marital status, educational attainment, personal income, employment status, year, and geographic location are also excluded from Table 1. A table reporting descriptive statistics for full set of variables is available from the authors on request.

AMI: acute myocardial infarction; CAD: coronary artery disease; CHF: congestive heart failure; HMO: health maintenance organization; NS: not significant; OOP: out of pocket; PC: prostate cancer

Table 2 presents the logistic regression estimates for the likelihood of incurring insurer and OOP expenditures. Patients in the localized cancer–no chemotherapy cohort had greater odds of incurring healthcare insurer expenditures (odds ratio [OR] 2.45, p<0.01). However, their odds of incurring increased OOP expenditures were not statistically significant (OR 1.41, p=0.15) Patients in the localized cancer with chemotherapy (severity 2) cohort and the metastatic (severity 3) patient cohort all incurred positive healthcare insurance expenditures. Hence, odds ratios of the variables of severity 2 and severity 3 were automatically excluded due to perfect prediction, meaning that all subjects with PC severity 2 and 3 had positive insurer expenditures. Patients with localized cancer treated with chemotherapy were substantially more likely to incur OOP expenditures compared to patients without PC (OR 5.89, p<0.01). The odds of incurring insurer and OOP expenditures were generally higher in all PC patients and among subjects who were 65 and over. African Americans, Hispanics, and patients with other race/ethnicity were less likely to incur healthcare insurer and OOP expenditures than were White patients.

The results in Table 2 were used to predict the likelihood of incurring healthcare insurer and OOP expenditures for each of the PC cohorts and the cohort without PC. Having PC raised the probability of incurring healthcare insurer expenditures in all three cohorts (Table 3). In the localized cancer without chemotherapy cohort the increase was 4 percentage points and in the other two cohorts the increase was 8 percentage points. Increases in the probability of OOP expenditures for the localized cancer without chemotherapy cohort was 1 percentage point, 6 percentage points for the localized cancer with chemotherapy cohort, and 4 percentage points for the metastatic cancer cohort.

Conditional regression models were also estimated for all three PC cohorts to determine the effects of PC severity on healthcare expenditures for patients incurring at least some positive expenditure. These models, reported in Table 4, included the same explanatory variables as in Table 2. The results indicated that health insurer and OOP costs increased significantly with PC severity.

The regression model results (Tables 2 and Table 4) were used to estimate expected incremental per-patient expenditures associated with PC for each cancer severity cohort. Having PC increased annual healthcare insurer expenditures by $5035, $15 895, and $19 794 for the three cohorts in increasing order of severity (Table 5). The presence of PC increased OOP expenditures by $204, $814, and $564 for the three cohorts in increasing order of severity (Table 5).

**Table 2.** Logistic Regression of the Probabilities of Positive Insurer Expenditure and Positive OOP Expenditures [per annum]

| | Total Insurer Cost >0 | | Total OOP Cost >0 | |
|---|---|---|---|---|
| | **Odds ratio** | **p-Value** | **Odds ratio** | **p-Value** |
| **Medical condition** | | | | |
| No PC | | Reference group | | |
| Localized PC - no chemotherapy | 2.45 | 0.01 | 1.41 | 0.15 |
| Localized PC - chemotherapy | - | | 5.89 | 0.00 |
| Metastatic PC | - | | 2.03 | 0.53 |
| **Medical condition (truncated)*** | | | | |
| **Diseases of the circulatory system** | | | | |
| Hypertension | 3.50 | 0.00 | 4.85 | 0.00 |
| CAD | 1.72 | 0.05 | 0.81 | 0.23 |
| CHF | 7.61 | 0.00 | 7.77 | 0.00 |
| AMI | 5.32 | 0.00 | 2.13 | 0.00 |
| Stroke | 9.05 | 0.03 | 11.41 | 0.01 |
| Cardiac dysrhythmias | 6.41 | 0.00 | 4.27 | 0.00 |
| **Diseases of the respiratory system** | | | | |
| Chronic bronchitis | 1.38 | 0.27 | 1.85 | 0.03 |
| Emphysema | 1.67 | 0.03 | 1.30 | 0.17 |
| Asthma | 3.41 | 0.00 | 3.73 | 0.00 |
| **Metabolic diseases** | | | | |
| Diabetes | 4.01 | 0.00 | 3.85 | 0.00 |
| Hyperlipidemia | 3.62 | 0.00 | 3.17 | 0.00 |
| Hypothyroidism | 4.21 | 0.00 | 7.95 | 0.00 |
| **Age** | | | | |
| 40–64 | | Reference group | | |
| 65–74 | 1.46 | 0.00 | 1.47 | 0.00 |
| ≥75 | 2.47 | 0.00 | 1.74 | 0.00 |
| **Race/ethnicity** | | | | |
| White | | Reference group | | |
| Hispanic | 0.73 | 0.00 | 0.67 | 0.00 |
| African American | 0.78 | 0.00 | 0.61 | 0.00 |
| Other | 0.61 | 0.00 | 0.56 | 0.00 |
| **Health insurance** | | | | |
| Private HMO plan | | Reference group | | |
| Medicaid | 1.76 | 0.00 | 0.50 | 0.00 |
| Medicare | 0.55 | 0.00 | 0.79 | 0.00 |
| Other public health insurance | 1.09 | 0.66 | 2.13 | 0.00 |
| Private non-HMO plan | 0.82 | 0.00 | 0.92 | 0.07 |
| **Sample size (N)** | 61 873 | | 62 359 | |
| Adj $R^2$ | 0.16 | | 0.16 | |

Note: The sample sizes are different. In the regression of Prob (total insurer cost), Localized PC - chemotherapy and Metastatic PC variables were dropped automatically, due to the perfect prediction (i.e. patients with Localized PC - chemotherapy and Metastatic PC all encountered third-party payment).

Table 2 footnotes, cont'd:

*In the interest of brevity, other generally less common conditions are not reported in Table 2. Variables measuring marital status, educational attainment, personal income, employment status, year, and geographic location are also excluded from Table 2. A table reporting the full set of logistic regression results is available from the authors on request.

AMI: acute myocardial infarction; CAD: coronary artery disease; CHF: congestive heart failure; HMO: health maintenance organization; OOP: out of pocket; PC: prostate cancer

**Table 3.** Effects of PC on Predicted Probability of Incurring Expenditures [per annum]

| | No PC | Localized PC – No chemotherapy | Localized PC – chemotherapy | Metastatic PC |
|---|---|---|---|---|
| Insurer expenditures | 0.92 | 0.96 | 1 | 1 |
| (Differences compared to no PC) | | 0.04 | 0.08 | 0.08 |
| OOP expenditures | 0.93 | 0.94 | 0.99 | 0.97 |
| (Differences compared to no PC) | | 0.01 | 0.06 | 0.04 |

OOP: out of pocket, PC: prostate cancer

**Table 4.** Ordinary Least Squares of Total Healthcare Insurer Expenditures and Total OOP Expenditures [per annum]

| | Total Insurer Cost (natural log) | | Total OOP Costs (natural log) | |
|---|---|---|---|---|
| | **Coefficient** | **p-Value** | **Coefficient** | **p-Value** |
| **Medical condition** | | | | |
| No PC | Reference Group | | | |
| Localized PC - No chemotherapy | 0.47 | 0.00 | 0.15 | 0.01 |
| Localized PC - Chemotherapy | 1.07 | 0.00 | 0.50 | 0.00 |
| Metastatic PC | 1.22 | 0.00 | 0.37 | 0.03 |
| **Medical Condition (truncated)*** | | | | |
| **Diseases of the circulatory system** | | | | |
| Hypertension | 0.37 | 0.00 | 0.38 | 0.00 |
| CAD | 0.24 | 0.00 | 0.08 | 0.01 |
| CHF | 0.77 | 0.00 | 0.36 | 0.00 |
| AMI | 0.87 | 0.00 | 0.38 | 0.00 |
| Stroke | 0.72 | 0.00 | 0.43 | 0.00 |
| Cardiac dysrhythmias | 0.66 | 0.00 | 0.39 | 0.00 |
| **Diseases of the respiratory system** | | | | |
| Chronic bronchitis | 0.20 | 0.01 | -0.13 | 0.05 |
| Emphysema | 0.29 | 0.00 | 0.12 | 0.01 |
| Asthma | 0.45 | 0.00 | 0.38 | 0.00 |
| **Metabolic diseases** | | | | |
| Diabetes | 0.58 | 0.00 | 0.51 | 0.00 |
| Hyperlipidemia | 0.40 | 0.00 | 0.38 | 0.00 |
| Hypothyroidism | 0.20 | 0.00 | 0.32 | 0.00 |

**Table 4.** Ordinary Least Squares of Total Healthcare Insurer Expenditures and Total OOP Expenditures [per annum] - cont'd

| | Total Insurer Cost (natural log) | | Total OOP Costs (natural log) | |
|---|---|---|---|---|
| | **Coefficient** | **p-Value** | **Coefficient** | **p-Value** |
| **Age (years)** | | | | |
| 40–64 | Reference group | | | |
| 65–74 | 0.17 | 0.00 | 0.18 | 0.00 |
| ≥75 | 0.36 | 0.00 | 0.26 | 0.00 |
| **Race/ethnicity** | | | | |
| White | Reference group | | | |
| Hispanic | -0.27 | 0.00 | -0.35 | 0.00 |
| African-American | -0.07 | 0.00 | -0.41 | 0.00 |
| Other | -0.23 | 0.00 | -0.38 | 0.00 |
| **Health insurance** | | | | |
| Private HMO plan | Reference group | | | |
| Medicaid | 0.46 | 0.00 | -0.92 | 0.00 |
| Medicare | -0.15 | 0.00 | 0.19 | 0.00 |
| Other public health insurance | -0.01 | 0.86 | 0.27 | 0.00 |
| Private non-HMO plan | 0.05 | 0.01 | 0.19 | 0.00 |
| **Constant** | 6.45 | 0.00 | 5.42 | 0.00 |
| **Sample size (N)** | 57 392 | | 57 637 | |
| Adj $R^2$ | 0.23 | | 0.20 | |

*In the interest of brevity, other generally less common conditions are not reported in Table 4. Variables measuring marital status, educational attainment, personal income, employment status, year, and geographic location are also excluded from Table 4. A table reporting the full set of ordinary least squares regression results is available from the authors on request.

AMI: acute myocardial infarction; CAD: coronary artery disease; CHF: congestive heart failure; HMO: health maintenance organization; OOP: out of pocket PC: prostate cancer

## Aggregate Expenditures and Sensitivity Analysis

US expenditures for PC for each severity cohort were calculated by multiplying the incremental expenditures in Table 5 with the estimated aggregate numbers of PC patients in each severity cohort. The aggregate number of PC patients in each severity cohort was determined by combining their respective prevalence rates estimated from MEPS with a US population estimate for males aged 40 and above, which was 67.5 million.[16] The baseline prevalence rate for PC was 1.33%, 0.73%, and 0.11% in the localized cancer not treated with chemotherapy, localized cancer treated with chemotherapy, and metastatic PC cohorts, respectively. These figures were consistent with ranges for PC reported in the literature.[15] The prevalence ranged from a low of 0.08% to a high of 1.66% in the sensitivity analysis based on the severity cohort. Baseline estimates of per-patient healthcare insurer and OOP expenditures from Table 5 varied plus or minus 10% for each prevalence level in the sensitivity analysis.

Aggregate US expenditures for PC by severity cohort are provided in Table 6. PC increased overall aggregate annual healthcare expenditures by $4.8 billion, $8.6 billion, and $1.6 billion for the localized PC with no chemotherapy, localized PC with chemotherapy, and metastatic PC cohorts, respectively. The overall total incremental expenditure for PC is thus estimated at $15 billion.

**Table 5.** Effects of PC on Expected Per-patient Healthcare Expenditures [per annum, 2011 dollars]

| | No PC | Localized PC—no chemotherapy | Localized PC—chemotherapy | Metastatic PC |
|---|---|---|---|---|
| Insurer expenditures | $8333 | $13 368 | $24 228 | $28 127 |
| (Differences compared to No PC) | | $5035 | $15 895 | $19 794 |
| OOP expenditures | $1264 | $1467 | $2078 | $1827 |
| (Differences compared to No PC) | | $204 | $814 | $564 |

Data shown in the above table are rounded to the nearest dollar. OOP: out of pocket; PC: prostate cancer

The sensitivity analysis demonstrated that annual healthcare expenditures ranged from $3.3 billion to $6.6 billion in the localized PC with no chemotherapy cohort. The ranges for the localized PC treated with chemotherapy and metastatic PC cohorts were $5.8 billion to $11.8 billion and $1.1 billion to $2.1 billion, respectively.

**Table 6.** Aggregate Expenditures and Sensitivity Analyses [$ billions per annum, 2011 dollars]

| **Localized PC—no chemotherapy ($ in billions)** | **Prevalence Rates** | | |
|---|---|---|---|
| Incremental effects | Low 1.0% | Base case 1.33% | High 1.66% |
| High (baseline +10%) | $4.0 | $5.3 | $6.6 |
| Base case | $3.6 | $4.8 | $6.0 |
| Low (baseline -10%) | $3.3 | $4.3 | $5.4 |

| **Localized PC—chemotherapy ($ in billions)** | **Prevalence Rates** | | |
|---|---|---|---|
| Incremental effects | Low 0.55% | Base case 0.73% | High 0.91% |
| High (baseline +10%) | $7.1 | $9.5 | $11.8 |
| Base case | $6.5 | $8.6 | $10.8 |
| Low (baseline -10%) | $5.8 | $7.7 | $9.7 |

| **Metastatic PC ($ in billions)** | **Prevalence Rates** | | |
|---|---|---|---|
| Incremental effects | Low 0.08% | Base case 0.11% | High 0.14% |
| High (baseline + 10%) | $1.3 | $1.7 | $2.1 |
| Base case | $1.2 | $1.6 | $2.0 |
| Low (baseline -10%) | $1.1 | $1.4 | $1.8 |

PC: prostate cancer

## Discussion

This study found that for patients with PC of all severity levels, their own OOP expenditures and the expenditures by their insurers were significantly higher than healthcare expenditures for similar individuals without PC. In adjusted analyses, the annual total incremental per patient healthcare expenditures ranged from a low of $5239 in the localized PC with no chemotherapy cohort to a high of $20 358 in the metastatic PC cohort (Figure 2). While the overall metastatic PC cohort's expenditures were higher than localized PC with chemotherapy cohort's, the OOP expenditures were lower ($564 vs. $814). It may be that patients with localized prostate cancer who received chemotherapy had higher OOP expenditures because their management involved types of care for which insurance coverage was less complete, such as higher and/or more co-pays for elective outpatient visits, physician visits, and over-the-counter medications.

**Figure 2.** Total incremental individual expenditures in 2011 US dollars

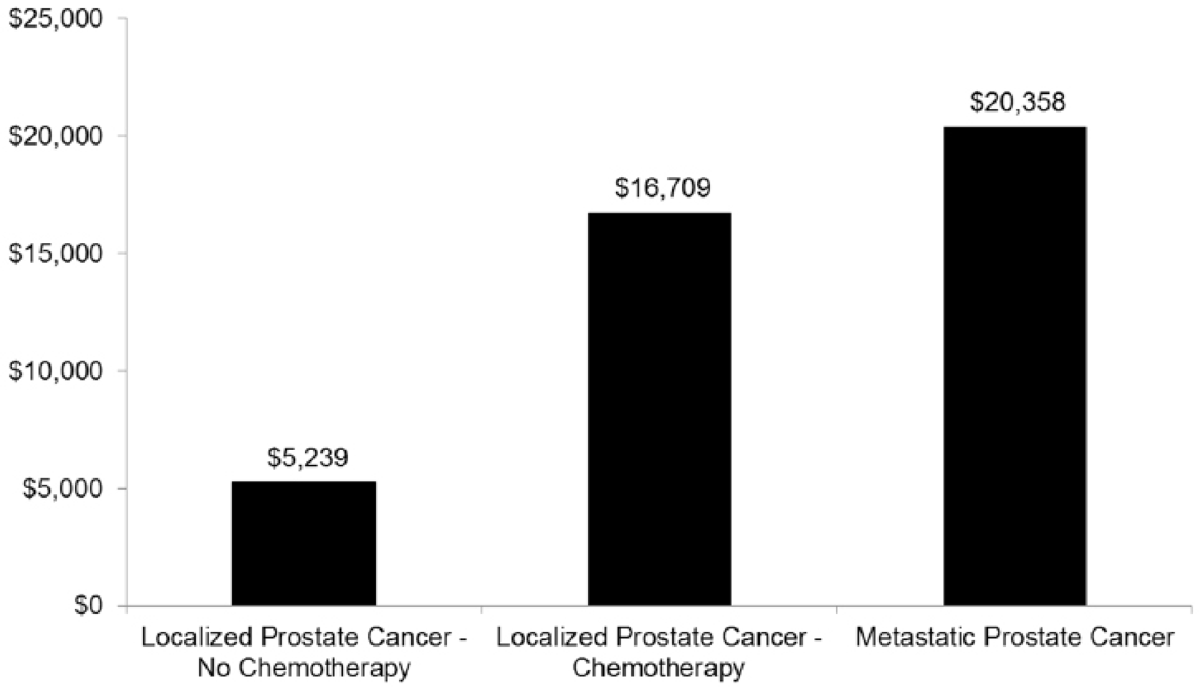

Aggregate costs by disease severity were largest for the localized PC cohort receiving chemotherapy ($8.6 billion annually), followed by localized PC with no chemotherapy ($4.8 billion) and metastatic PC ($1.6 billion). The large aggregate costs of PC—$15 billion annually—suggest that interventions such as early detection, as well as new and more effective treatments for this disease, may confer important economic as well as clinical benefits. Any such economic evaluation would need to compare the costs of the intervention against potential cost savings and clinical benefits due to that treatment.

Despite the high occurrence and costs associated with PC, these patients often face disparities in funding, awareness, media coverage, and research compared to other equally common cancers, contributing to fewer treatment options and worse outcomes.[17] For instance, a 2007 study by the US-based National PC Coalition found that there were seven breast cancer drugs on the market for every PC drug.[17]

There are also disparities in detection, with governments offering less financial support and fewer mandates for PC screening in comparison to breast cancer. Indeed, a 2007 report noted that 49 US states mandated insurance coverage for routine breast cancer screening, but only 28 had similar mandates for PC screening.[17] There is also significantly less media coverage for PC relative to other prevalent cancers. A study by the Prostate Coalition found that for each story in the media covering PC, there were 2.6 stories on breast cancer.[17]

This study is the first to provide nationally representative estimates of the direct healthcare expenditures for PC by disease severity. Prior studies included populations that may not have been generalizable or did not stratify by severity of disease.[5,7,8,18,19] The design of MEPS helps ameliorate this problem by employing a sample that is nationally representative. By including a sensitivity analysis using a range of PC prevalence estimates, this study addressed the tendency for prevalence rates to be underreported in surveys, a major methodological challenge recognized in survey research.[20] In addition, this study included 30 relevant comorbid diseases, which helped control for potential confounding effects. Finally, the two-part model methodology is a widely accepted method of estimating expenditures in the econometrics literature because it adjusts for patients who have no expenditures.[13,21] If this adjustment is not made, estimates of incremental healthcare expenditures associated with a disease are less reliable.[13,21] Most studies of healthcare expenditures in patients with PC have not used this model.[5,7,8,19] We also gained onsite access to the MEPS restricted files rather than using the publicly available standard MEPS data. This allowed the research team to obtain more detailed ICD-9 code information, which improved stratification of disease severity.

## LIMITATIONS

This study has important limitations that must be noted. First, relevant factors that are related to healthcare expenditures may have been omitted. However, given that the study included 30 comorbidities and a number of socio-demographic factors, the effect of any omissions may be attenuated. Second, this study was based on self-reported data collected as part of a national household survey, which has the potential of recall bias. MEPs design addressed this issue by collecting data from physician and insurance records in addition to the patient reports. The reported disease prevalence may not accurately reflect actual disease prevalence. This issue was attenuated by performing a sensitivity analysis that varied the prevalence of PC. Finally, we measure disease severity using a proxy measure because the data source employed in this analysis did not provide the necessary granularity of information to clinically assign cancer disease stage. Moreover, the MEPS database does not include information such as whether a patient received radiation treatment or underwent prostatectomy, which would have permitted more detailed delineation of PC severity. However, with knowledge of secondary malignancies and chemotherapy drug agents being used, we were able to segment the PC population by those with taking or not taking chemotherapy drug agents and metastatic cancer.

## CONCLUSION

PC is a significant public health concern in the United States and the most prevalent cancer among US men. The aggregate costs of PC are substantial for all disease severity groups examined, and are greatest for patients with localized cancer treated with chemotherapy. This reflects the relatively high prevalence and high per capita healthcare expenditures associated with this cohort. Per capita healthcare expenditures are greatest for the metastatic PC cohort, however. With a growing and aging population, the prevalence of PC is expected to rise, further increasing the public health burden of the disease. Future studies should continue to analyze shifting patterns in the clinical and economic burden of PC to provide a more informed basis for health policy decision making.

## ACKNOWLEDGMENTS

The authors wish to acknowledge CTI Clinical Trial and Consulting Services for analyzable dataset creation, analysis, statistical, and writing support. Writing and editorial support were provided by Jennifer DiNieri, Matt Dougherty, and Lisa Sullivan at StemScientific, an Ashfield Company, funded by Bristol-Myers Squibb. The publication of study results was not contingent on the sponsor's approval or censorship of the manuscript.
